# Socio-economic inequalities in rates of amenable mortality in Scotland: Analyses of the fundamental causes using the Scottish Longitudinal Study, 1991–2010

**DOI:** 10.1002/psp.2385

**Published:** 2020-09-22

**Authors:** Megan A. McMinn, Rosie Seaman, Ruth Dundas, Jill P. Pell, Alastair H. Leyland

**Affiliations:** 1 MRC/CSO Social and Public Health Sciences Unit, University of Glasgow, Glasgow, UK; 2 Faculty of Social Sciences, University of Stirling, Stirling, UK; 3 Institute of Health and Wellbeing, University of Glasgow, Glasgow, UK; 4 Usher Institute, University of Edinburgh, Edinburgh,UK

**Keywords:** amenable mortality, socio-economic inequality, Fundamental Causes Theory, Scotland

## Abstract

Socio-economic inequalities in amenable mortality rates are increasing across Europe, which is an affront to universal healthcare systems where the numbers of, and inequalities in, amenable deaths should be minimal and declining over time. However, the fundamental causes theory proposes that inequalities in health will be largest across preventable causes, where unequally distributed resources can be used to gain an advantage. Information on individual-level inequalities that may better reflect the fundamental causes remains limited. We used the Scottish Longitudinal Study, with follow-up to 2010 to examine trends in amenable mortality by a range of socioeconomic position measures. Large inequalities were found for all measures of socioeconomic position and were lowest for educational attainment, higher for social class and highest for social connection. To reduce inequalities, amenable mortality needs to be interpreted both as an indicator of healthcare quality and as a reflection of the unequal distribution of socio-economic resources.

## 1 Introduction

Despite the existence of a universal healthcare system in the United Kingdom, a socio-economic gap in health has persisted (Steel & Cylus, 2012). Health inequalities in Scotland are of particular concern, with the gap in all-cause mortality rates between the most and least deprived areas increasing since 1981, owing to greater declines in mortality among the less deprived areas than experienced by the most deprived (Leyland, Dundas, McLoone, & Boddy, 2007). In response, the Scottish Government’s Ministerial Task Force on Health Inequalities recommended the monitoring of inequalities in premature all-cause mortality as an indicator of the country’s progress in reducing health inequalities (Scottish Government, 2008). Mortality amenable to healthcare intervention (amenable mortality) is an extension to this indicator, measuring unnecessary premature deaths (Rutstein et al., 1976).

Amenable mortality was initially introduced as a measure of the quality of medical care delivered, as deaths resulting from the identified conditions could be avoided, given timely and effective healthcare (Nolte & McKee, 2004). Since being introduced, changes in rates of amenable mortality over time and differences between countries have been explored. Socio-economic gradients in rates of amenable mortality have also been found with rates of amenable mortality being highest for the most disadvantaged groups and lowest for the least disadvantaged groups (Lumme, Manderbacka, Karvonen, & Keskimäki, 2018; Mackenbach et al., 2017).

The strong relationship between socio-economic inequality and amenable mortality demonstrates that it is not simply a measure of medical care quality. Instead amenable mortality reflects the unequal distribution of socio-economic resources that continue to play a role in determining health and enable individuals to navigate healthcare. Therefore, it is imperative that we begin to understand which dimensions of socio-economic inequality are most important for avoiding amenable deaths, especially in the context of effective and universal healthcare systems where the numbers of, and inequalities in, amenable deaths should be minimal and should be declining over time.

This paper forms part of a special issue on the ‘Social Inequalities in Health and Mortality: The Analysis of Longitudinal Register Data from Selected European Countries’. Improving upon the existing evidence for socio-economic inequalities in amenable mortality in Scotland, we have examined a wider range of conditions considered to be amenable to healthcare intervention, using the Scottish Longitudinal Study (SLS). We have repeated our analyses for four individual level measures of socio-economic inequality: two measures of social class, educational attainment and social connection. We have quantified and compared absolute and relative inequalities in amenable, non-amenable and all-cause mortality for each of the four individual-level measures of socio-economic inequality by calculating indices of inequality. The use of two Census periods, with four harmonised measures of socio-economic inequality, provided a unique opportunity to identify any changes over time to inequalities in amenable mortality in Scotland, a country with some of the starkest mortality inequalities in Europe, despite having a universal and effective healthcare system.

## 2 Background

### 2.1 Fundamental causes theory and amenable mortality

The theory of fundamental causes argues that socio-economic inequalities in health and mortality will continue to persist because the resources, which are embodied in socio-economic status, can be used to protect health (Link & Phelan, 1995; Phelan, Link, & Tehranifar, 2010). Socio-economic groups make use of some, or all, of their disposable resources to negate health risks, to adopt protective strategies and ultimately avoid poor health and an untimely death. Within the fundamental causes theory, resources can be both material, such as money and immaterial, such as power, prestige, knowledge or advantageous social connections. Individuals from the most disadvantaged socio-economic groups may lack these resources or be in a position which does not empower them to use their resources explicitly to improve their health, even when there is a universal and effective healthcare system, which places an emphasis on prevention (Mackenbach et al., 2015; Lumme et al., 2018; Mackenbach et al., 2017).

Due to the unequal distribution of resources, it is implicit to the fundamental causes theory that inequalities in mortality will be largest across causes of death which are preventable and smallest across nonpreventable causes, where resources cannot be used to gain an advantage. In support of this theoretical perspective, relative inequalities were found to be largest for causes amenable to behavioural changes, and to a lesser extent, for causes amenable to healthcare intervention and injury prevention. Relative inequalities for causes deemed not-preventable were indeed smallest (Mackenbach et al., 2015). This finding was consistent across 19 European countries. Further studies have demonstrated gradients in amenable mortality across individual-level dimensions of socio-economic inequality, including occupation (Blakely, 2001; Mackenbach, Stronks, & Kunst, 1989), education (Stirbu et al., 2010) and marital status (Manderbacka, Peltonen, & Martikainen, 2014). These are measures of socio-economic inequality that are likely to capture some of the different resources described by the fundamental causes theory.

### 2.2 Measures of socio-economic inequality and fundamental causes resources

#### 2.2.1 Social class

The first identification of socio-economic inequalities in amenable mortality was measured using Social Class based on Occupation in England and Wales. Greater relative declines in amenable mortality rates were identified for the higher classes, compared to the lower classes, for men and married women (Mackenbach et al., 1989). Occupations reflect social standing and may relate to health in general through autonomy in workplace, differing reward structures and privileges and work-place exposures such as physical labour or toxic exposures (Geyer & Peter, 2000; Shaw & Galobardes, 2007). Occupation-based social classes are one of the more commonly used measures of socio-economic position in the United Kingdom, where a range of measures have been developed. However, similar occupational class gradients in avoidable mortality have been found in other countries (Blakely, 2001; Poikolainen & Eskola, 1995; Wood, Sallar, Schechter, & Hogg, 1999). The first official measure of social class in the United Kingdom was the Registrar General’s Social Classes (RGSC; later known as Social Class based on Occupation) was used between 1911 and 2000 (Shaw & Galobardes, 2007). It comprised five ordinal categories and ranged from unskilled to professional occupations, with the third category divided between skilled manual and nonmanual jobs. Assignments were based on job title, and the RGSC has therefore been criticised for having a weak theoretical basis (Rose & Pevalin, 2003). RGSC measure was superseded by the National Statistics Socioeconomic Classification (NS-SEC) in the 2000s. The NS-SEC was not designed to be better than the Social Class based on Occupation, rather to improve the possibility of explaining statistical associations. However, the NS-SEC included additional information such as company size, job security, prospects of promotion and level of autonomy in order to classify an individual into one of 14 nonhierarchical operational classes (Rose & Pevalin, 2003). NS-SEC measures conditions of employment and occupational relations. Using this more theoretical-based measure means, it can be considered a better reflection of the types of socio-economic resources highlighted by the fundamental causes theory.

#### 2.2.2 Educational attainment

Educational attainment is favoured in studies exploring mortality inequalities, as it typically remains stable from early adult life, and is considered less prone to reverse causation (Stirbu et al., 2010). Educational attainment is associated with the types of resources that are required to improve health generally but that may also be key for navigating the healthcare system to reduce the risk of amenable death specifically, such as greater health awareness and health literacy (Bautista, Alfonso, Corella, & Saiz, 2005). Previous analyses using education data from 16 European countries, once again support a fundamental causes perspective as relative inequalities in rates of amenable mortality exceeded those estimated for all-cause mortality, with significant differences found in 11 countries (Stirbu et al., 2010).

#### 2.2.3 Social connection

A positive relationship between higher levels of social connection and better health has been widely documented (Hughes & Waite, 2002; Umberson, Crosnoe, & Reczek, 2010) and is explicitly outlined in the fundamental causes theory. Marital status can be considered an indicator of social connection as individuals who are married or in a relationship are assumed to have higher levels of social support and shared economic advantages compared with single people (Lund et al., 2002). Being in a relationship may enable both individuals to engage and navigate healthcare more effectively. This is evident in relation to amenable deaths as hazard ratios were found to be lowest for those living in an unmarried cohabiting couple and highest for those living alone, in comparison with married couples (Manderbacka, Peltonen, et al., 2014). Although the ratios were attenuated after adjustment for other measures of socio-economic inequality (education, income and economic activity), the difference between the levels of social connection remained statistically significant, emphasising that social connection is an important resource for health. In terms of general health, marital status has often been found to have stronger, positive effect for men than for women (Robards, Evandrou, Falkingham, & Vlachantoni, 2012).

#### 2.2.4 Individual-level linked data

An important feature of the majority of studies of amenable mortality is that most have used individual-level linked data. This means that the measures of socio-economic inequality used were often constructed by linking individual-level socio-economic data and death data on unique personal identifiers that are available for the whole population (Lumme, Sund, Leyland, & Keskimäki, 2012; McCallum, Manderbacka, Arffman, Leyland, & Keskimäki, 2013; Stirbu et al., 2010). This is important as individual level measures of socio-economic inequality are more likely to capture the types of resources highlighted within the fundamental causes theory. Within the United Kingdom, data are somewhat limited as population-wide unique personal identifiers do not exist for record linkage beyond healthcare registers. Studies of amenable mortality in the United Kingdom have therefore been carried out using unlinked study designs, which are known to be prone to numerator/denominator biases (Tobias & Cheung, 2003), and area-level measures of deprivation which are based on alternative theoretical frameworks (Kearns, Gibb, & Mackay, 2000). However, large-scale linkage studies, which combine administrative, healthcare registers and vital events data sources for a large representative sample of the U.K. population are increasingly available. The SLS is one such study.

Replicating analyses conducted in Europe on population wide, individual-level linked data (Mackenbach et al., 2008), Popham and Boyle (2010) used educational attainment data from the SLS to examine amenable mortality in Scotland. Absolute inequalities in rates of amenable mortality in Scotland were found to be amongst the highest in the selected European countries, exceeded only by Lithuania and Estonia for men. However, Popham and Boyle (2010) only examined the gradient in amenable mortality using one measure of socio-economic inequality, level of educational qualification.

Although education is likely to reflect resources such as knowledge, health awareness and health literacy (Bautista et al., 2005) other measures of socio-economic inequality may capture alternative socioeconomic resources which may be more important for minimising the risk of amenable death. Identifying differences in the direction or magnitude of inequality in amenable mortality, across a number of socio-economic measures, may help to better identify which resources.

## 3 Data and Methods

### 3.1 The SLS

The SLS provided the opportunity to investigate the effects of individual-level measures of socio-economic inequality on amenable mortality within Scotland over 20 years for a large representative sample of the population. The SLS comprised a number of administrative, health register and vital events data, including Census responses (first linked in 1991), the National Health Service Central Register (NHSCR, health data) and Vital Events (births, deaths and marriages), for a 5.3% sample of the Scottish population (Hattersley & Boyle, 2007). New data from subsequent Censuses are linked to the sample in the years following a Census. Vital events are usually linked on an annual basis (Hattersley & Boyle, 2007). The socio-economic information from the Census returns and linked to SLS members, deemed most relevant for this research, were two derived measures of occupation-based social class, educational attainment and marital status and household structure, which we combined to create a measure of social connection.

### 3.2 SLS study members

Study members are included in the sample if they were born on one of 20 semirandom birth dates, in each calendar year. To link study members to NHS health data, the members must be traced in the NHSCR, meaning they must have been registered with the NHS. Therefore, the SLS sample includes both persons born in Scotland and those who have immigrated to Scotland, provided they have registered with an NHS primary healthcare centre. Entries and exits from the study are tracked through registrations and deregistrations from the NHS, as well as birth and death records (Boyle et al., 2009).

Completion of the Census and the registration of vital events are compulsory in Scotland. As a result, at least 96% of the SLS sample have been traced and stored in the database (Boyle et al., 2009; Hattersley & Boyle, 2008; Hattersley, Raab, & Boyle, 2007). Due to the sensitive and confidential nature of the SLS, it is only accessible from a secure setting.

### 3.3 Study outcome

The main outcome of interest was deaths amenable to healthcare intervention, with secondary outcomes of nonamenable deaths and all-cause. These outcomes were identified in the SLS sample through record-linkage to the vital events datasets.

#### 3.3.1 Amenable, nonamenable and all-cause deaths

The conditions considered to be amenable to healthcare intervention in this analysis were a combination of five of the most commonly used or recently published lists of amenable conditions (Lumme et al., 2012; McCallum et al., 2013; Nolte & McKee, 2004, 2008; Page et al., 2006). This means our study included the most comprehensive coverage of amenable conditions published to date (see Appendix A). The extent to which deaths from Ischaemic Heart Disease can be avoided through healthcare intervention has previously been contested (Nolte & McKee, 2004, 2011). Two of the lists used to inform our list of conditions considered 50% of IHD deaths to be amenable. We decided against this approach as the proportion was unlikely to be uniform across different socio-economic groups in Scotland (Davies, Dundas, & Leyland, 2009). The remaining three lists either analysed IHD deaths separately, or excluded them completely. We chose to exclude Ischaemic Heart Disease from our list of amenable conditions.

Nonamenable deaths were comprised of all remaining deaths which were not considered to be amenable to healthcare intervention and included Ischaemic Heart Disease. Amenable deaths and nonamenable deaths were mutually exclusive categories. All-cause mortality was comprised of both amenable and non-amenable deaths.

### 3.4 Study exposures and additional covariates

Four individual level measures of socio-economic inequality that could be derived from the 1991 and 2001 Censuses were included as study exposures: two measures of occupation-based social class derived from occupational data, educational attainment, and whether the member lived as part of a married or cohabiting couple, or not (social connection). All four measures were harmonised across the two Censuses to ensure comparability.

#### 3.4.1 Social class

Two measure of occupation-based social class were available in the SLS database: Social Class based on Occupation and the NS-SEC (Rose & Pevalin, 2003). The NS-SEC was introduced in 2001 and was designed to supersede Social Class based on Occupation, however both measures were calculated for each Census allowing for comparisons between the two measures.

Social Class based on Occupation was designed to be an ordinal measure, comprised of 6 categories: (1) professional, (2) managerial and technical, (3N) skilled nonmanual and (3M) skilled manual, (4) partly skilled and (5) unskilled occupations. The NS-SEC is made up of 14 operational categories of occupation, which are not ordinal. The statistical methods used in the analyses (slope index and relative index of inequality) required categories to be ordered in increasing levels of disadvantage. Therefore, the 14 categories were collapsed into five categories for analyses: (1) managerial and professional, (2) intermediate, (3) lower supervisory and technical, (4) semiroutine and (5) routine occupations. This was based upon the recommended three classes, with the third class (routine and manual occupations) split into three further categories in order to explore the more disadvantaged socio-economic groups. In both measures, the long-term unemployed, students and those otherwise economically inactive were not assigned to a category; therefore, they are not included in the analyses for the two social class measures only.

#### 3.4.2 Educational attainment

Levels of educational attainment were defined according to the categories used for the 1991 census and comprised (1) first and higher degree, (2) other higher qualification (nondegree level) and (3) no over 18 qualification. The 2001 Census allowed for a more detailed breakdown of the categories; however, to ensure comparability, the 2001 variable was collapsed to the three levels described.

#### 3.4.3 Social connection

The measure of socio-economic inequality explored was whether the SLS member lived in the same household as part of a married or cohabiting couple compared with SLS members who were not married or part of a cohabiting couplet. This measure aimed to capture differences in the level of social connection. Combining marital status and cohabiting individuals into one category reflects the growing body of contemporary demographic evidence which shows that nonmarital cohabitation and marriage are linked (Sassler & Lichter, 2020). Having coresident children has previously been shown to not negatively impact the health of parents (Hughes & Waite, 2002). At the same time, more recent evidence suggests that having adult children, transition back to a parental home may have particularly negative effects on mental health (Caputo, 2019). Given that the main outcome measure of interest was amenable mortality, the decision was made to assign SLS to either category of social connection regardless of whether they had any children living with them or not. Same sex marriage has been legal in Scotland since December 16th 2014. In the 2011 Census, there was the option for individuals to self-report that they were part of a same sex civil partnership. The last recorded data we used for our measures of social connection was from the 2001 Census. Therefore, our measure of social connection was constructed from self-reports of marriage or cohabitation only which were likely to include same sex couples.

#### 3.4.4 Additional covariates

In addition to the socio-economic inequality measure of interest, we also included sex (men and women) and five-year age group (35–74 years).

### 3.5 Sample population

The SLS sample consisted of 306,771 members at the time of analyses. These include SLS members of any age, identified in the 1991 and 2001 Census returns, as well as those linked through the NHSCR in the 10 year period following each Census. At the time of submitting our application to use the SLS database, Census records for 1991, 2001 and 2011 were linked for SLS members; however, only vital events up to 2010 had been linked to SLS members. This meant that it was only possible to use the socio-economic inequality data from the 1991 and 2001 Censuses linked with the mortality data from 1991 through to the end of 2010.

#### 3.5.1 Sample population inclusion criteria

To be included in the sample population as a numerator or denominator, the SLS member had to have been enumerated at the previous Census (1991, 2001 or both), and aged between 35 and 74 years. This upper age limit is widely accepted as the upper limit for defining an amenable death (Nolte & McKee, 2004). The lower age limit for amenable deaths in current literature tends to be birth; however, for this analyses, we increased it to age 35 years in order to use the most relevant individual-level measures of socio-economic inequality. By this age, the majority of members will be in employment, have completed their education and are more likely to be in a relationship., Therefore socio-economic measures at each Census were considered to be more stable and meaningful than those reported at earlier ages, or those which are derived from parental socio-economic data. As a result of the age criteria, an SLS member had to have been aged 26 years in 1991, to contribute towards the numerator and/or denominator by 2000, when they turned 35 years old. Rates of overall mortality before the age of 35 years are relatively low in Scotland. In addition, overall rates of amenable mortality at the youngest ages are low. Therefore, the lower age limit of 35 years is unlikely to have had a large effect on our results. The analyses of all-cause and nonamenable deaths were also restricted to ages 35–74 years.

We extracted all deaths held in the SLS Vital Events dataset. Deaths occurring between 21 April 1991 (Census day) and 31 December 2000, and between 29 April 2001 (Census day) and 31 December 2010 (inclusive), and at ages 35 to 74 years are included in the analysis. Deaths occurring between 1 January and 28 April 2001 were excluded in order to match the approximate time at risk available for the first Census period (21 April 1991–31 December 2000).

Within the two analysis periods (excluding 1 January–28 April 2001) there were 40,307 sample member deaths at all ages, of which 22,757 (56%) occurred between the ages of 35 and 74 years. Of these, 5,964 (15% of all deaths and 26% of deaths between 35 and 74) were classified as being amenable to medical care. The remaining deaths (74%) were classified as nonamenable deaths.

#### 3.5.2 Sample population exclusion criteria

As a Census is a self-completed questionnaire, there is a risk of item nonresponse in one or more of the responses required to construct the measures of socio-economic inequality, or additional covariates. We removed data for 85 (0.03%) SLS members from all further analyses due to having a nonresponse recorded for sex, year of birth, or having conflicting years of birth and ages at the corresponding Census. Where there was a missing, or unclassifiable response for a piece of information required to measure socio-economic inequality, the individual was excluded from the count of deaths (nominator) and person-years at risk (denominator) for that specific measure of socio-economic inequality. This means that the numbers of deaths and person-years at risk were not constant across all four measures of socio-economic inequality but that the sample available for each measure of socio-economic inequality was maximised. The missing or unclassifiable responses were more prevalent in the occupation-based social class measures, as they required multiple answers in order to be assigned to an occupational category. The educational attainment and social connection measures were each derived from a single question.

### 3.6 Methods

#### 3.6.1 Age-standardised mortality rates and person-years at risk

Sex-specific age-standardised mortality rates per 100,000 person-years were calculated separately for amenable, nonamenable and all-cause mortality for each of the two Census time periods, using the 2013 European Standard Population (European Commission, 2013). Person-years at risk were used as the denominator and were calculated for the whole sample over two periods, using the relevant Census date as the start date for each period. The person-years at risk were adjusted for emigration from Scotland and death and aggregated to 5-year age groups. Therefore, each member could contribute a maximum of 5 years ‘at risk’ within each age group to the denominator (Flanagan & McCartney, 2015; Harding, 1995). The person-years at risk were calculated for 306,686 SLS members, of whom 49% were male and 51% were female.

#### 3.6.2 Indices of inequality

For each measure of socio-economic inequality, the relative inequality between mortality rates of the notionally most advantaged and least advantaged group was calculated using the relative index of inequality (RII), modelled using Poisson regression (Mackenbach et al., 2008). Numbers of deaths from amenable causes, nonamenable causes and all-causes were modelled separately as the outcome, with the person-years at risk used as the offset. The inclusion of a population fraction variable within the Poisson model means that the RII takes the whole socio-economic distribution in the socio-economic inequality indicator in the population into account. This acts as a continuous ranking value for each level of socio-economic inequality within the model and improves over a simple relative or absolute difference estimation, which only uses the two extreme levels. This ranking variable represents the cumulative distribution of the population of that variable, and is therefore sensitive to changes in the distributions of measures of socio-economic inequality within the study population over time (Moreno-Betancur, Latouche, Menvielle, Kunst, & Rey, 2015; Popham & Boyle, 2010). The RII assumes that rates of mortality increase with increasing disadvantage. An RII of 1 suggests no inequality in mortality across the socio-economic gradient, whereas an RII of 2 is interpreted as the mortality rate in the notionally most disadvantaged group being double that of the notionally least disadvantaged, after having taken the whole socio-economic gradient into account.

The equivalent inequality measure on the absolute scale is the slope index of inequality (SII; Mackenbach et al., 2008), derived as

$$SII=2\times age\;adjusted\;mortality\times \frac{\left(RII-1\right)}{\left(RII+1\right)}$$

The SII describes the absolute difference in mortality rates between the notionally most and least advantaged groups of each socio-economic inequality measure, defined here as deaths per 100,000 person-years (Mackenbach et al., 2008).

The RII and SII were calculated for amenable mortality, non-amenable mortality and all-cause mortality for each combination of sex, Census period and measure of socio-economic inequality. Narrowing of both relative and absolute inequalities between Census periods would require that the most disadvantaged socio-economic group experienced greater absolute and relative declines in their mortality rates, compared to the higher levels, whereas smaller declines would lead to widening inequalities (Mackenbach et al., 2016). The widening of one inequality index does not necessarily imply that the other will have similarly widened. Therefore the absolute and relative results are presented in order to provide a full illustration of inequalities in mortality by each measure of socio-economic inequality (Scottish Government, 2020) and is in line with best practice (King, Harper, & Young, 2012).

Monte Carlo simulation was used to simulate 95% confidence intervals around each RII estimate (Lumme, Sund, Leyland, & Keskimäki, 2015), and the above equation was also used to transform the confidence intervals around the SII estimate. The inequality indices have been used in many settings and were recommended for use in measuring the inequality gradient in Scotland (Scottish Government, 2008).

## 4 Results

### 4.1 Percentage of amenable and nonamenable deaths

Table 1 shows the percentage of deaths defined as amenable or non-amenable, the numbers of all-cause deaths (rounded to nearest 10), and associated person-years at risk for the four measures of socio-economic inequality. The educational attainment and social connection measures contain the largest numbers of all-cause deaths, and person-years at risk, whereas the two measures of social class have the smallest numbers.

For men and women, the percentage of deaths, which were defined as amenable, decreased or stayed stable over time. This finding was evident across all measures of socio-economic inequality. However, in both Census periods, amenable deaths accounted for a higher percentage of deaths among women than deaths among men: approximately 30% of all-cause deaths among women compared with approximately 20% of all-cause deaths among men.

### 4.2 Changes to amenable mortality over time

Detailed graphs showing sex-specific mortality rates, comparing the two Census periods, and for each measure of socio-economic inequality are given in Appendix B. For both men and women, amenable, nonamenable and all-cause mortality rates were lowest for the least disadvantage groups and highest for the most disadvantage. This finding was consistent for the two measures of social class, education attainment and our measure of social connection. Although a general gradient was evident across all four measures of socio-economic inequality, there was not always a consistent step-wise increase in mortality rates with each increase in level of disadvantage.

#### 4.2.1 Social class

For men, amenable mortality rates consistently decreased between the two Census periods for each occupational group, this was evident when measuring occupation-based social class measures and the NS-SEC. However, very few decreases were statistically significant as the confidence intervals for the two Census period overlap for males from almost all of the occupation groups (occupation-based social class and NS-SEC). For women, amenable mortality rates did not consistently decrease between the two Census periods and some occupation groups were found to have slightly higher amenable mortality rates in 2001–2010 than in 1991–2000. This was evident when using occupation-based social class and the NS-SEC. However, the confidence intervals for amenable mortality rates among women in the two Census periods overlap for every occupation group. Some confidence intervals for women are very wide, such as professional occupations, which is due to small numbers. The same general findings for amenable mortality are evident when looking at nonamenable and allcause mortality by each social class group.

#### 4.2.2 Educational attainment

Between the two Census periods, amenable mortality rates decreased for both men and women from all educational attainment categories. However, these decreases were only statistically significant for the lowest educational group (no over 18 qualification), as confidence intervals did not overlap. The decreases in amenable mortality rates for the other educational groups are not statistically significant as the confidence intervals did overlap. The same patterns were evident when looking at nonamenable and all-cause mortality by educational attainment.

#### 4.2.3 Social connection

In both Census periods, amenable mortality, nonamenable mortality and all-cause mortality were significantly lower for individuals defined has having a higher level of social connection (individuals who self-reported that they were married or in a cohabiting couple). This was true for men and women. Over time there were statistically significant declines in amenable, nonamenable and all-cause mortality rates for both categories of our social connection measure.

### 4.3 Relative and absolute inequalities

Table 2 shows the RII results, which quantified the relative inequality in mortality rates between the notionally highest and lowest categories of each measure of socio-economic inequality, whilst taking into account the full distribution, rather than just the two extreme categories. Table 3 shows equivalent absolute inequalities in mortality rates, calculated using the SII.

In all but one measure of socio-economic inequality (educational attainment) relative inequality for amenable, nonamenable and all-cause mortality increased between the two census periods. Increases found for all-cause mortality were significant but increases in relative inequality for amenable and nonamenable were not consistently significant for men and women, across all measures of socio-economic inequality. The only significant increases for amenable mortality were for women, using the occupation-based measure of social class and the measure of social connection.

The magnitude of relative inequality for amenable mortality, non-amenable mortality, and all-cause mortality among men tended to exceed the magnitude of relative inequality among women, regardless of the measure of socio-economic inequality.

The SII for amenable mortality rates were smaller than the SII for nonamenable mortality rates and all-cause mortality rates. This was the case for both men and women. However, this result was expected given the calculation of the SII is dependent on the mortality rate.

The following sections summarise the key RII and SII findings for each measure of socio-economic inequality, paying attention to changes over time and any notable gender differences.

#### 4.3.1 Social class

The magnitude of relative inequality for amenable, nonamenable and all-cause mortality was similar within the two measures of social class (Table 2). However, the RII estimated for occupation-based social class tended to be lower than the RII estimated for NS-SEC for men in the first Census period but there was little difference between the RII estimates in the second Census time period. For women, the magnitude of relative inequality was very similar between the two measures of social class.

Relative inequality increased over time for amenable, non-amenable and all-cause mortality for men and women, when using the two measures of social class (Table 2). For example, when measuring occupation-based social class, the RII for amenable mortality among men in 1991–2000 was 1.8 (95% CI [1.5, 2.3]). This means that men from the lowest occupation-based social class category had an amenable mortality rate 1.8 times that of the highest category. By 2001–2010, the amenable mortality rate for men from the lowest category had increased and was 2.5 (95% CI [2.0, 3.2]) times higher.

Absolute inequalities in amenable, nonamenable and all-cause mortality increased when measured using the two occupational social class measures for both sexes (Table 3). However, most of the confidence intervals overlapped between the two Census periods, meaning that most of these increases in absolute inequality were not significant.

The only statistically significant increase over time in absolute inequality was for all-cause mortality among men when measuring occupation-based social class. The absolute inequality in all-cause mortality rates between men from the lowest occupation category and men from the highest occupation category was 744 per 100,000 person-years (95% CI [636, 851]) in 1991–2000. This increased to 935 per 100,000 (95% CI [856,1,011]) in 2001–2010.

#### 4.3.2 Educational attainment

The magnitude of relative inequality for amenable, nonamenable and all-cause mortality were similar when socio-economic inequality was measured using educational attainment (Table 2).

When measuring social inequality by educational attainment, the RII for amenable mortality among men in the period 1991–2000 was 3.7 (95% CI [2.6, 5.6]). This means that men with the lowest levels of education had rates of amenable mortality 3.7 times those with the highest levels of education, a finding that is statistically significant as the confidence intervals do not include the null value of one. During the period 2001–2010, there was a slight decrease in the RII for amenable mortality to 3.6. For men, relative inequality in nonamenable and all-cause mortality also decreased over time when measured using educational attainment.

For women, a decrease in relative inequality by educational attainment was only evident for amenable mortality. Nonamenable and all-cause mortality by educational attainment increased for women between the two Census time periods, but the increase was not statistically significant as the confidence intervals overlap.

The absolute inequality in amenable mortality rates between men with the lowest level of education and men with the highest levels of education was 361 per 100,000 person-years (95% CI [276, 440]; Table 3). This decreased to 245 per 100,000 (95% CI [194, 293]) in the following census period but was not a statistically significant decrease as the confidence intervals overlap.

#### 4.3.3 Social connection

For both men and women, relative inequality in amenable mortality, nonamenable mortality and all-cause mortality increased between the two Census time periods when measured using our indicator of social connection (Table 2). For men notable differences between the magnitudes of RII estimates for amenable, nonamenable and all-cause mortality rates were found when using the social connection measure: Relative inequalities in amenable mortality were much larger compared with relative inequalities in nonamenable and all-cause mortality. A particularly striking finding is the magnitude of relative inequality in amenable mortality for men when measuring social connection compared to women: The RII estimates for men were double that of the estimates for women.

Again, there were striking results for changes to absolute inequality over time for amenable, nonamenable and all-cause mortality when measuring social connection. Absolute inequality decreased over time between men categorised as having higher social connection and men categorised as having lower social connection. For women, the opposite pattern was found and absolute inequality increased for amenable, nonamenable and all-cause mortality when measuring social connection.

## 5 Discussion

### 5.1 Summary of main results for amenable mortality

Inequality in amenable mortality was evident in Scotland across all four measures of socio-economic inequality, with the most disadvantaged group experiencing higher rates of amenable mortality than the least disadvantaged group. When looking at changes over time, the magnitude of inequalities in amenable mortality did not consistently increase or decrease across all four measures of socio-economic inequality used.

For the two measures of social class, absolute and relative inequality in amenable mortality widened over time. Widening inequalities occur when the notionally least disadvantaged experience greater declines in mortality rates, compared to the notionally most disadvantaged. Therefore, men and women in the highest social class categories experienced greater improvements in mortality rates than the lowest social class categories.

In contrast, absolute and relative inequality in amenable mortality narrowed when looking at educational attainment. Narrowing inequalities occur when the notionally least disadvantaged experience smaller declines in mortality rates, compared with the notionally most disadvantaged. Therefore, those with the lowest level of educational experienced greater improvements in mortality rates, compared to those with the highest level of educational attainment.

When investigating the impact of social connection on amenable mortality, opposing patterns were found between relative and absolute inequality. Relative inequalities widened over time for men and women. Absolute inequalities also widened, but for women only. Narrowing absolute inequalities for men means that there were greater absolute improvements in mortality rates for men who were categorised as having lower levels of social connection (not married or not in a cohabiting relationship) compared with men categorised as having higher levels of social connection (married or cohabiting relationship).

### 5.2 Theoretical reflections

The efficient and universal healthcare system available in Scotland should ensure that relative inequalities in rates of amenable mortality are smaller than relative inequalities in all-cause mortality, irrespective of which measure of socio-economic inequality is used. We found no significant differences in the magnitude of relative inequality for amenable mortality compared with the magnitude of relative inequality for all-cause mortality. For men in particular, relative inequality tended be very similar, or even higher for amenable deaths, compared to the all-cause deaths.

When considering the fundamental causes perspective, it is not unexpected to find that inequalities in amenable mortality were larger than all-cause mortality. In contrast to the notion that amenable mortality is reduced by the healthcare system, fundamental causes theory argues that socio-economic resources are actually *more* important for reducing the risk of dying from an amenable cause than they are for reducing the risk of dying from causes which cannot be avoided through interventions offered by the healthcare system.

We examined four different measures of socio-economic inequality, over two Census time periods, in an attempt to identify if either social class, education or social connection were more important for amenable mortality in Scotland. The four measures of socio-economic inequality were selected as potential indicators of the types of socio-economic resources, highlighted as important for health by the fundamental causes theory. Expanding on the original fundamental causes theory, Phelan et al. (2010) identified four components of evidence which research should test in relation to the fundamental causes theory (1) that socio-economic inequality influences multiple disease outcomes; (2) that socio-economic inequality is related to multiple risk factors for disease and death; (3) that the deployment of resources plays a critical role in the association between socio-economic inequality and health/mortality; and (4) that the association between socio-economic inequality and health/mortality is reproduced over time via the replacement of intervening mechanisms. Although we found clear evidence that amenable mortality is patterned by socio-economic inequality, over time and across multiple indicators of socio-economic inequality, this is not enough to fully support all four components of the fundamental causes theory. The deployment of resources and the replacement of mechanisms are particularly interesting directions for future research into amenable mortality. This may be better achieved by identifying populations where there is no socio-economic inequality in amenable mortality and identify how the ability to use socio-economic resources to gain a health advantage is blocked. An alternative approach would be to examine the impact of any new treatments for causes of death included in the amenable mortality category, as the fundamental causes theory hypothesised that the introduction of new treatments can lead to new mechanisms which enable socio-economic resources to be used to gain an advantage.

Alongside the theoretical limitations of our study, it is important to reflect on the empirical data and methodological decisions that may have had an impact on our results.

### 5.3 Data and methodological considerations

Our measures of socio-economic inequality, derived from Census responses, assumed that individuals remained in the same socio-economic group over each interim 10-year period. This assumption may be reasonable for educational attainment, given the lower age limit of 35 years in the study population inclusion criteria. However, social class category and marital or cohabitation status are more likely to fluctuate for many individuals over a 10-year period (Boyle et al., 2009). If we take educational attainment to be the most stable indicator of socio-economic inequality for our study population then the results for social class and social connection may be either under-estimations or overestimations, depending on the direction of change individuals experienced. Although it would have been possible to identify individual changes in self reports of education, social class, and social connection only 63% of SLS members, age 35–74 years old, were identified in the 1991 Census and the 2001 Census.

All four measures of socio-economic inequality were captured from the same two Census years. However, each measure is likely to have a different meaning depending on the age of each individual. For the oldest members of the study population, the level of education attained over 50 years ago may have less relevance for their current socio-economic resources but social connection may play an increasingly important role for maintaining their health. In contrast, educational attainment might provide more valuable resources for health for the youngest members of the study population, as they are yet to reach their own, most meaningful social class position based on their occupation.

The numbers of deaths and person-years at risk were not the same for each measure of socio-economic inequality. A large proportion of deaths were excluded from the two social class measures, with only 66% of deaths among men and 53% of deaths among women under the age of 75 being assigned to the occupation-based social class measure. The corresponding proportions were 65% and 52% for the NS-SEC measure. It was only possible to assign the economically active population to the two measures of social class. This means that the unclassified members of the study population were likely to include some of the most disadvantaged groups in society, such as the long-term unemployed, never employed, those with intermittent casual work, or those with disabilities or impairments which do not allow them to participate in work due to a lack of workplace adaptions (Office for National Statistics, 2015). Therefore, our results for social class measures were likely to have been subject to health selection bias, whereby only those healthy enough to be in employment are categorised. Students were also excluded from the social class measures. However, given the lower age limit of 35 years, students would have only account for a small minority of the unclassified group.

Another issue which contributed to the lower numbers of deaths and person-years for the two social class measures is that they required multiple questions within the Census to be answered in order to be assigned to a category. In contrast, educational attainment and cohabitation status can be ascertained from one question. The potential impact of the lower number of classifiable deaths, and person-years at risk, for the social class measures is difficult to predict but it is likely that results are only generalizable to the economically active population. The findings for the educational attainment and social connection measures were applicable to the wider Scottish population.

The SLS is a large, nationally representative sample of the Scottish population, with a high percentage of members linked across two Censuses. However, the data we used relied on the accurate linkage of Census returns to death certificates. It is possible that the SLS Vital Events dataset, through which the deaths to SLS members are recorded and linked, was not complete as a result of delayed registrations of deaths, or unlinked deaths due to date of birth discrepancies (Popham & Boyle, 2010). This is unlikely to have an effect on the results presented here, as the probability of unlinked deaths was not related to socio-economic inequality. Changes in the composition of the categories in each socio-economic measure within the study population were unlikely to affect the results as this is controlled for with the inclusion of a population fraction within the Poisson model used to estimate the RII.

The choice of conditions identified as amenable can be perceived as judgement based, and we acknowledge that the division between amenable and nonamenable conditions is not exact (Nolte & McKee, 2004). However, our analyses have provided a comprehensive description of inequalities in rates of deaths from conditions which are broadly considered to be treatable in the context of the National Health Service. The conditions considered to be amenable to healthcare intervention were collated from five of the most recently published, or widely used lists (Lumme et al., 2012; McCallum et al., 2013; Nolte & McKee, 2004, 2008; Page et al., 2006) and was the most up-to-date measure of amenable mortality at the time of research.

Previous research into amenable mortality divided the amenable conditions into three further categories: deaths amenable to (1) primary prevention; (2) early detection and intervention; and (3) improved treatment and medical care (Lumme et al., 2012). These categories would allow for the identification and monitoring of areas within a healthcare system which are producing the largest mortality rates or are responsible for the greatest inequalities. The numbers of deaths which occurred within the study population were insufficient to allow for further exploration, without the results being strongly impacted by random fluctuations.

Our analyses only included SLS linked data for the 1991 and 2001 Census. The 2011 Census is currently linked to SLS members and more recent measures of socio-economic inequality are now available. However, at the time of analyses, deaths were only linked up to the end of 2010 (now linked to end of 2013). Once the linkage of more recent deaths has been performed, it will be possible to estimate inequalities using the 2011 Census measures of socio-economic inequality compared to those presented in this paper.

A wider range of measures of socio-economic inequality could be considered for future analyses of amenable mortality using the SLS, such as ethnicity and income. Unfortunately for our analyses there were insufficient numbers in this sample to explore ethnicity. Approximately 84% of the study population in 2001, who had subsequently died of an amenable condition, had self-reported as being White. The majority of the remaining 16% of amenable deaths had no self-reported information on ethnicity. Therefore, the numbers were too small to draw any valid conclusions, especially once deaths were split by sex and socio-economic measures. Data from other sources, such as the SHELS study (Bhopal et al., 2010) or other methods of statistical analysis may better support an exploration of the relationship between of ethnicity and amenable mortality. Income is not collected in the Census, however, methods to estimate synthetic income measures for SLS members have been developed (Clemens & Dibben, 2014), which could allow for income inequalities in mortality to be investigated. Expanding our understanding of multiple measures of socio-economic inequality may reveal further differences within and between amenable and nonamenable mortality in Scotland. Perhaps a more important research direction for the future is to examine the intersection of dimensions of socio-economic inequality and whether there is an accumulation or buffering effect of each on mortality risk (Hill, 2015). For example, in Finland, unemployment did not increase the risk of amenable deaths in the presence of high income and not living alone. However, low income was found to increase the risk of amenable death among those employed and cohabiting (Manderbacka, Arffman, Sund, & Karvonen, 2014).

### 5.4 Conclusion

Numbers of amenable deaths should be minimal, and there should be little to no socio-economic gradient, given that access to prevention, detection, and treatment services, which are capable of averting amenable deaths, are free and universal in Scotland. In reality, equitable access to healthcare is unlikely to completely reduce inequalities in amenable deaths, as health is influenced by a range of socio-economic factors beyond the healthcare system.

Taking a fundamental causes perspective, we examined four different measures of socio-economic inequality. Each of the four measures of socio-economic inequality aimed to capture different resources that were likely to be important for reducing the risk of an amenable death: Social class, educational attainment, and social connection. The persistence of inequalities in amenable mortality across all measures of socio-economic inequality indicates that health improvements, facilitated only by the healthcare system, are likely to have greater effects on those in more advantaged groups. If the healthcare system is seen as the only means for reducing amenable mortality, it is likely that inequalities in amenable mortality will widen in both relative and absolute terms.

## Supplementary Material

Appendix

## Figures and Tables

**Table 1:** Percentage of amenable and nonamenable deaths and number of all cause deaths with associated person-years at risk for persons with each reported measure of socio-economic inequality, ages 35 to 74 years, for 1991–2000^a^ and 2001–2010^b^

	Amenable (%)	Nonamenable (%)	All cause (*N*)	Person-years at risk (*N*)
All SLS member deaths				
Men				
1991–2000	23	77	7,230	567,189
2001–2010	22	78	5,830	593,352
Women				
1991–2000	33	67	5,310	615,965
2001–2010	31	69	4,400	648,165
Occupation-based social class				
Men				
1991–2000	21	79	4,960	507,700
2001–2010	21	79	4,690	549,400
Women				
1991–2000	34	66	2,280	444,030
2001–2010	31	69	3,320	585,345
NS-SEC				
Men				
1991–2000	21	79	4,970	509,941
2001–2010	21	79	4,460	530,119
Women				
1991–2000	34	66	2,270	441,210
2001–2010	31	69	3,190	565,282
Educational attainment				
Men				
1991–2000	22	78	6,810	548,770
2001–2010	21	79	5,150	564,989
Women				
1991–2000	32	68	4,930	587,827
2001–2010	31	69	3,780	615,066
Social connection				
Men				
1991–2000	22	78	7,050	562,273
2001–2010	21	79	5,420	570,279
Women				
1991–2000	33	67	5,180	612,406
2001–2010	31	69	4,090	620,714

*Note*. Source: Scottish Longitudinal Study.

a 21 April 1991–31 December 2000.

b 29 April 2001–31 December 2010.

c All cause deaths are rounded to nearest 10.

**Table 2:** Relative Indices of Inequality (95% CI) for the four individual-level measures of socio-economic inequality for amenable and nonamenable causes, and all causes, ages 35 to 74 years,1991–2000^a^ and 2001–2010^b^

	Amenable	Nonamenable	All cause
Occupation-based social class			
Men			
1991–2000	1.84 (1.48, 2.29)	1.92 (1.71, 2.15)	1.90 (1.72, 2.10)
2001–2010	2.51 (2.01, 3.15)	2.94 (2.63, 3.31)	2.85 (2.57, 3.16)
Women			
1991–2000	1.52 (1.18, 1.96)	1.80 (1.50, 2.17)	1.70 (1.47, 1.97)
2001–2010	2.52 (2.01, 3.19)	2.22 (1.90, 2.59)	2.31 (2.04, 2.62)
NS-SEC			
Men			
1991–2000	2.03 (1.63, 2.56)	2.04 (1.82, 2.29)	2.04 (1.85, 2.26)
2001–2010	2.68 (2.12, 3.41)	2.76 (2.45, 3.11)	2.74 (2.47, 3.05)
Women			
1991–2000	1.49 (1.15, 1.94)	1.94(1.61,2.35)	1.78 (1.52, 2.06)
2001–2010	2.45 (1.94, 3.13)	2.26 (1.93, 2.66)	2.32 (2.03, 2.65)
Educational attainment			
Men			
1991–2000	3.68 (2.55, 5.61)	3.64 (2.97, 4.55)	3.65 (3.05, 4.40)
2001–2010	3.57(2.61, 5.12)	3.23 (2.73, 3.85)	3.30 (2.84, 3.85)
Women			
1991–2000	2.41 (1.69, 3.57)	2.63 (2.02, 3.48)	2.56 (2.05, 3.20)
2001–2010	2.29 (1.68, 3.21)	2.77 (2.22, 3.49)	2.61 (2.17, 3.16)
Social connection			
Men			
1991–2000	3.31 (2.66, 4.09)	2.55 (2.27, 2.87)	2.71 (2.44, 3.00)
2001–2010	4.85 (3.72, 6.31)	3.71 (3.24, 4.25)	3.92 (3.48, 4.43)
Women			
1991–2000	1.51 (1.24, 1.84)	1.86 (1.61, 2.13)	1.74(1.55, 1.95)
2001–2010	2.30 (1.76, 3.00)	2.75 (2.28, 3.29)	2.59 (2.24, 3.01)

*Note*. Source: Scottish Longitudinal Study.

a 21 April 1991–31 December 2000.

b 29 April 2001–31 December 2010.

**Table 3:** Slope Indices of Inequality (95% CI) for the four individual-level measures of socio-economic inequality, for amenable and nonamenable causes, and all causes, ages 35 to 74 years, 1991–2000^a^ and 2001–2010^b^

	Amenable	Nonamenable	All Cause
Occupation-based social class			
Men			
1991–2000	140.2 (91.6, 185.9)	594.4 (497.4, 689.5)	743.7 (635.8, 851.2)
2001–2010	164.5 (128.4, 198.1)	758.3 (690.1, 825.4)	934.8 (856.4, 1010.6)
Women			
1991–2000	85.1 (34.3, 133.7)	260.3 (182.3, 335.9)	351.1(258.1,441.9)
2001–2010	160.0 (123.9, 193.6)	339.0 (278.5, 395.3)	510.0 (441.7, 576.5)
NS-SEC			
Men			
1991–2000	171.9 (120.7, 220.7)	647.4 (550.9, 741.2)	819.3 (712.8, 925.1)
2001–2010	184.1 (144.3, 220.2)	709.4 (636.2, 778.9)	893.6 (812.1, 972.1)
Women			
1991–2000	87.5 (30.9, 141.8)	291.7 (212.5, 367.2)	378.2 (279.0, 469.5)
2001–2010	164.9 (125.4, 202.1)	342.1 (279.7, 400.6)	507.4 (434.4, 576.8)
Educational attainment			
Men			
1991–2000	361.2 (275.8, 440.1)	1249.0 (1088.3, 1402.9)	1610.1 (1431.0, 1779.1)
2001–2010	244.6 (193.7, 292.6)	841.8 (740.1, 937.8)	1086.1 (973.0, 1194.3)
Women			
1991–2000	233.4 (145.3, 317.2)	528.8 (398.6, 652.5)	762.0 (599.8, 912.4)
2001–2010	164.1 (106.1, 219.7)	437.8 (352.3, 516.8)	601.3 (499.5, 701.1)
Social connection			
Men			
1991–2000	335.7 (284.1, 380.2)	961.3 (854.0, 1061.8)	1301.1 (1181.4, 1412.4)
2001–2010	293.4 (256.8, 323.8)	939.2 (862.6, 1011.2)	1234.3 (1150.3, 1313.3)
Women			
1991–2000	115.2 (60.5, 166.4)	351.3 (275.0, 421.9)	468.0 (373.1, 557.4)
2001–2010	165.7(116.2,210.6)	447.1 (373.5, 512.1)	612.1 (527.5, 692.8)

*Note*. Source: Scottish Longitudinal Study.

a 21 April 1991–31 December 2000.

b 29 April 2001–31 December 2010.
