# Supplementary material for: Socio-economic inequalities in rates of amenable mortality in Scotland: Analyses of the fundamental causes using the Scottish Longitudinal Study, 1991–2010
Source: Popul Space Place. Author manuscript; Available in PMC 2022 Apr 10. (PMC7612592; doi:10.1002/psp.2385)
Supplement: Appendix [file EMS137984-supplement-Appendix.pdf]

APPENDIX A

TABLE A1 Causes of death considered to be amenable to health care intervention, with age limits and ICD 9 and 10 code, by subgroups

Cause of death	Age	ICD-9	ICD-10
Primary prevention			
Intestinal Infections	0-14	001-009	A00-A09
Other infections (diphtheria, tetanus, poliomyelitis and varicella)	0-74	032, 037, 045, 052	A35, A36, A80, B01
Whooping cough	0-14	033	A37
Scarlatina	0-74	034.1	A38
Meningococcus	0-74	036	A39
Erysipelas	0-74	035	A46
Measles	0-14	055	B05
Rubella	0-74	056	B06
Malaria	0-74	084	B50-B54
Streptococcalpharyngitis	0-74	034	J02.0
Cellulitis	0-74	681-682	L03
Early detection and intervention			
Tuberculosis	0-74	010-018, 137	A15 - A19, B90
Malignant neoplasm of colon and rectum	0-74	153, 154	C18-C21
Melanoma of skin	0-74	172	C43
Malignant neoplasm of skin	0-74	173	C44
Malignant neoplasm of breast	0-74	174	C50
Malignant neoplasm of cervix uteri	0-74	180	C53
Malignant neoplasm of unspecified parts of uterus and body of uterus	0-44	179, 182	C54, C55
Malignant neoplasm of bladder	0-74	188	C67
Neoplasm of Thyroid	0-74	193	C73
Benign tumours	0-74	210-229	D10-D36
Hypertensive disease	0-74	401-405	I10-I13, I115
Cerebrovascular disease	0-74	430-438	I60-I69
Bacterial Meningitis	0-74	320	G00, G03
Improved treatment and medical care			
Septicaemia	0-74	038	A40, A41
Legionelliosis	0-74	482.84	A48.1
Malignant neoplasm of testis	0-74	186	C62
Hodgkin's disease	0-74	201	C81
Leukaemia	0-44	204-208	C91-C95
Diseases of the thyroid	0-74	240-246	E00-E07
Diabetes mellitus	0-74	250	E10-E14
Epilepsy	0-74	345	G40,G41
Improved treatment and medical care continued			
Rheumatic and other valvular heart disease	0-74	390-398	I01-I09
Nephritis and nephrosis	0-74	580-589, 591	N00-N08, N17-N19
All respiratory diseases (excluding pneumonia / influenza)	0-14	460-478, 494, 495, 500-519	J00-J06, J20-J22, J30-J39, J47-J99
Influenza	0-74	487, 488	J09-J11
Pneumonia	0-74	480-486	J12-J18
Chronic obstructive pulmonary disease	45-74	490-492,496	J40-J44
Asthma	0-44	493	J45, J46

(Continues)

TABLE A1 (Continued)

Cause of death	Age	ICD-9	ICD-10
Peptic ulcer	0-74	531-534	K25-K28
Appendicitis	0-74	540-543	K35-K38
Abdominal hernia	0-74	550-553	K40-K46
Cholelithiasis and cholecystitis	0-74	574-575.1	K80-K81
Other diseases of the gallbladder	0-74	575.2-575.9	K82
Other diseases of the billary tract	0-74	576	K83, K91.5
Diseases of pancreas	0-74	577	K85,K86
Obstructive uropathy and prostatic hyperplasia	0-74	592, 593.7, 594, 598.0-598.1, 598.8-598.9, 600	N13, N20-N21, N35, N40
Maternal death	0-74	630-676	O00-O99
Perinatal deaths (all causes excluding stillbirths)	ALL	760-779	P00-P03, P05-P95, A33, A34
Congenital cardiovascular anomalies	0-74	745-747	Q20-Q28
Misadventure to patients during surgical and medical care	0-74	E870-E876, E878-E879	Y60-Y69, Y83-Y84

APPENDIX B

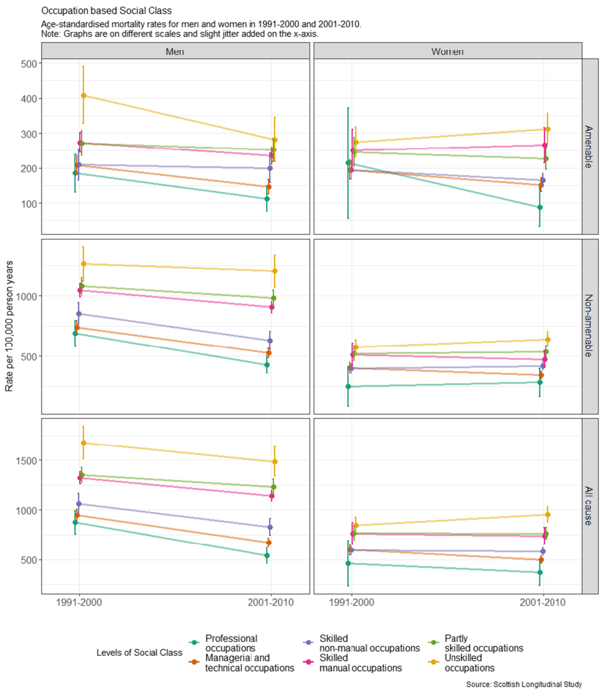

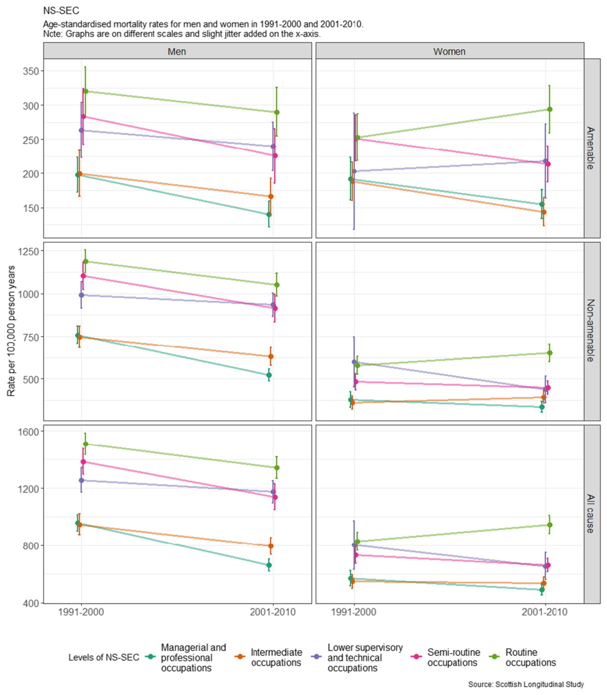

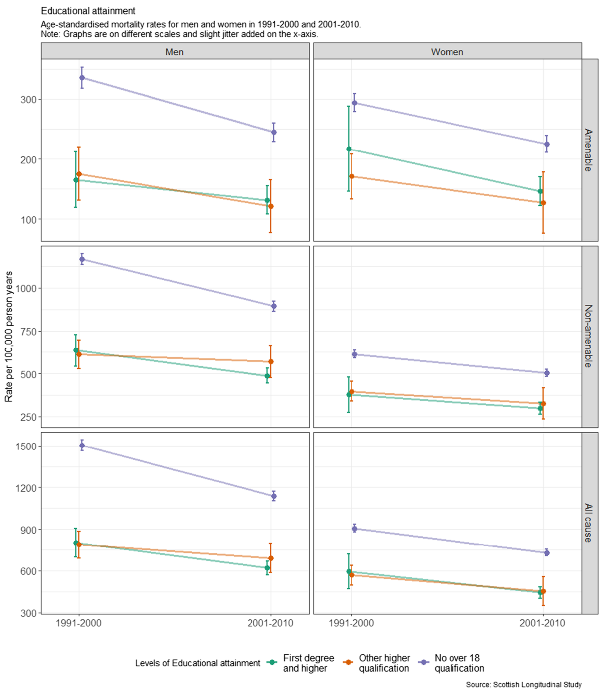
Educational attainment
Age-standardised mortality rates for men and women in 1991-2000 and 2001-2010.
Note: Graphs are on different scales and slight jitter added on the x-axis.
Men
Women
Amenable
Non-amenable
All cause
Rate per 100,000 person years
1991-2000
2001-2010
Levels of Educational attainment
First degree and higher
Other higher qualification
No over 18 qualification
Source: Scottish Longitudinal Study

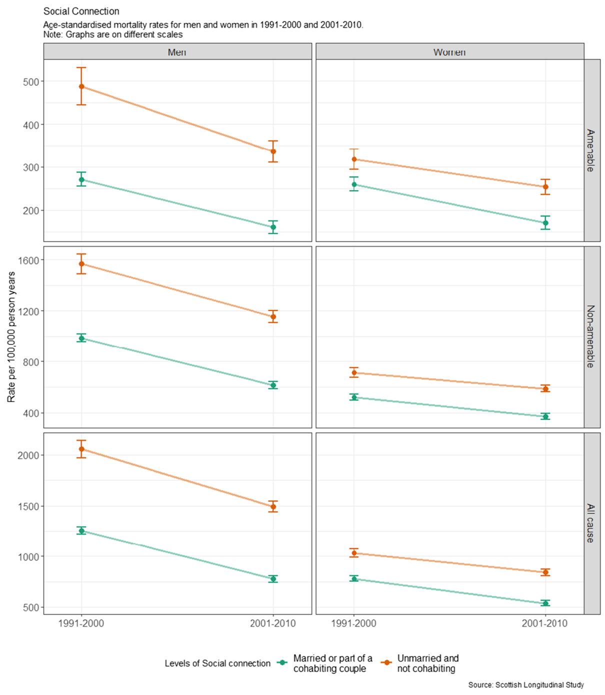
Social Connection
Age-standardised mortality rates for men and women in 1991-2000 and 2001-2010.
Note: Graphs are on different scales
Men
Women
Amenable
Non-amenable
All cause
Rate per 100,000 person years
1991-2000
2001-2010
Levels of Social connection
Married or part of a cohabiting couple
Unmarried and not cohabiting
Source: Scottish Longitudinal Study
